# Computational Biology and Machine Learning Approaches to Understand Mechanistic Microbiome-Host Interactions

**DOI:** 10.3389/fmicb.2021.618856

**Published:** 2021-05-11

**Authors:** Padhmanand Sudhakar, Kathleen Machiels, Bram Verstockt, Tamas Korcsmaros, Séverine Vermeire

**Affiliations:** ^1^Department of Chronic Diseases, Metabolism and Ageing, Translational Research Center for Gastrointestinal Disorders (TARGID), KU Leuven, Leuven, Belgium; ^2^Earlham Institute, Norwich, United Kingdom; ^3^Quadram Institute Bioscience, Norwich, United Kingdom; ^4^Department of Gastroenterology and Hepatology, University Hospitals Leuven, KU Leuven, Leuven, Belgium

**Keywords:** health, disease, microbiome-host interactions, molecular mechanisms, computational approaches, machine learning, basic and clinical research

## Abstract

The microbiome, by virtue of its interactions with the host, is implicated in various host functions including its influence on nutrition and homeostasis. Many chronic diseases such as diabetes, cancer, inflammatory bowel diseases are characterized by a disruption of microbial communities in at least one biological niche/organ system. Various molecular mechanisms between microbial and host components such as proteins, RNAs, metabolites have recently been identified, thus filling many gaps in our understanding of how the microbiome modulates host processes. Concurrently, high-throughput technologies have enabled the profiling of heterogeneous datasets capturing community level changes in the microbiome as well as the host responses. However, due to limitations in parallel sampling and analytical procedures, big gaps still exist in terms of how the microbiome mechanistically influences host functions at a system and community level. In the past decade, computational biology and machine learning methodologies have been developed with the aim of filling the existing gaps. Due to the agnostic nature of the tools, they have been applied in diverse disease contexts to analyze and infer the interactions between the microbiome and host molecular components. Some of these approaches allow the identification and analysis of affected downstream host processes. Most of the tools statistically or mechanistically integrate different types of -omic and meta -omic datasets followed by functional/biological interpretation. In this review, we provide an overview of the landscape of computational approaches for investigating mechanistic interactions between individual microbes/microbiome and the host and the opportunities for basic and clinical research. These could include but are not limited to the development of activity- and mechanism-based biomarkers, uncovering mechanisms for therapeutic interventions and generating integrated signatures to stratify patients.

## Introduction: Microbiome-Host Interactions

Across different niches and ecosystems, micro-organisms including bacteria, viruses, archaea inhabit a wide range of hosts (Braga et al., 2016). This community of microbes imparts various functions such as making nutrients accessible to the host (Martin et al., 2019), modulating the host immune system (Mendes et al., 2019), warding off pathogens (Pickard et al., 2017), maintaining homeostasis (Ohland and Jobin, 2015; Penny et al., 2018) among others. These functions are in turn driven primarily by molecular interactions between microbial and host molecules such as proteins, RNA and metabolites (Hughes and Sperandio, 2008; Braga et al., 2016). Deciphering these interactions could not only reveal the microbe-host cross-talk but also provide us with insights into formulating therapeutic strategies aimed at maintaining health and/or ameliorating disease states. The past decades have witnessed a surge in research interest to study microbial communities (and their interactions) which inhabit various niches – from the gut to the soil ecosystem. This was made possible by technological advancements leading to plummeting costs of 16S and metagenomic sequencing, higher sequencing depth and resolution (Levy and Myers, 2016; Jacob et al., 2019; Valli et al., 2020), novel *in vitro* systems (Shah et al., 2016; Eain et al., 2017; May et al., 2017), and new methodologies for high-throughput profiling of multiple -omic data types such as metaproteomics, metabolomics, lipidomics (Muller et al., 2013; Roume et al., 2015). However, due to many other limitations related to scale, scope, feasibility and sample availability for parallel omic read -outs, experimentally determining the inter-species microbe-host interactions is a challenging task (Fritz et al., 2013). Computational methods can overcome some of these limitations thereby enhancing our understanding of microbe-host interactions (Dix et al., 2016). In this review, we outline some key concepts, tools, and methods involved in computationally inferring the molecular mechanisms mediating microbe-host interactions.

## Biological Networks: Concepts and Applications

Biological networks represent relationships (termed edges) between any two biological entities (species, organisms, and molecules, etc.) which are usually called as nodes. At the level of molecules (genes, proteins, metabolites, RNAs, and small molecules, etc.), biological networks could either denote the physical interactions (e.g., protein–protein, protein-DNA, and RNA-protein, etc.) between molecules or any measure of association (e.g., co-expression and co-occurrence) between molecules (Gosak et al., 2018). In this paper, we will refer only to physical interactions. Physical interactions can be classified based on various criteria such as molecular types (protein–protein, protein-DNA, and RNA-protein, etc.), experimental scale (high-throughput or low-throughput), source (experimentally determined or computationally predicted), directionality (directed or undirected), relational signs (positive or negative relationships) and coverage (genome-wide or targeted). Since biological networks provide the larger context in which genes or proteins tend to exert their action, researchers can thereby fine-tune their hypotheses. Networks have largely been used in the domain of biological sciences (a) as a scaffold to integrate either singular or multiple contextual -omic datasets such as gene expression, proteomics, etc., measured in response to intrinsic or extrinsic stimuli (Charitou et al., 2016), (b) as a graph to trace potential signaling and regulatory pathways connecting any two nodes (Azeloglu and Iyengar, 2015), (c) to perform functional analysis at a local or global level (Emmert-Streib and Glazko, 2011), (d) to reconstruct the networks of non-model organisms from those of model organisms (Thompson et al., 2015), (e) to discover drug and disease targets (Huang et al., 2018), and (f) to infer globally or locally conserved signatures such as modules, motifs, etc (Wong et al., 2012). Various resources of molecular interactions and tools for integrative network analysis have been compiled and developed by the research community of network biologists. Since a very detailed description of the resources and tools is out of scope of the current review, readers are hereby referred to Pedamallu and Ozdamar (2014), Miryala et al. (2018), Romano et al. (2019).

Due to their utility in capturing contextual backgrounds and communication between molecular entities, biological networks have been used to not only study intra-species interactions but also inter-species cross-talks. Molecular ecological networks (Deng et al., 2012; Heleno et al., 2014) are a case in point by which the concept of networks are used to study the interactions between molecules (derived from different species or even kingdoms) in a larger ecological context (Yang et al., 2017; Meyer et al., 2020; Yu et al., 2020; Zheng et al., 2020). At the very core of it, a typical molecular ecological network inference workflow (Zhou et al., 2010; Deng et al., 2012; Chen et al., 2017) starts with the generation of meta -omic datasets (such as metagenomics, metatranscriptomics, and metaproteomics, etc.) followed by differential abundance testing between samples from contrasting conditions. Various measures of correlations and associations can then be applied to determine the distance between samples based on the differences and similarities in terms of the molecular features measured in the -omic datasets across the sample classes. Such correlations or associations can be used as a primary point of reference to investigate the possibility of mechanistic interactions which could in turn be driving the associative relationships. Furthermore, a network based representation of the feature-space can be used to compare samples with each other or to associate network properties such as the presence of motifs and modules to higher-level ecological traits/phenotypes. However, since molecular ecological networks do not directly infer molecular mechanisms which is the topic of this review, a detailed discussion on the topic is not undertaken.

## Computational Methods in Microbiome-Host Interactions: Filling the Gaps

Computational methods bring in various advantages to the analysis of interactions between the host and individual microbes and/or the microbial community. These include their attributes of (a) enhancing scalability, i.e., perform the computational inferences for a large number of variables and samples, (b) improving reproducibility (if complemented by inter-operability, automation, proper version control and sufficient documentation), (c) assessing performance by using a series of metrics, (d) shortlisting and prioritizing interactions, (e) and thereby (f) enabling the fine-tuning of hypothesis for experimental and/or epidemiological studies. Although most of the methods hitherto have focused on inferring the interactions between individual microbial species (mostly well studied pathogens) and the host, a few methods have been developed to predict the interactions at a community level. In principle, many of the methods which have been used to infer interactions of single species can be scaled up (with appropriate modifications) to infer community level interactions.

## Classification of Computational Methods in Microbiome-Host Interactions

**FIGURE 1:**
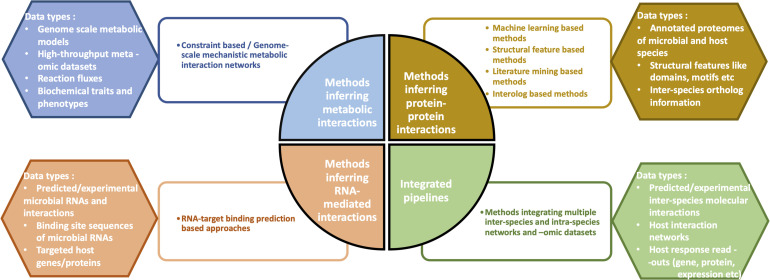
Overview of the four different categories of computational methods which help infer the molecular mechanisms of microbe-host interactions. Some examples of data types corresponding to each of the four methods are depicted.

From a mechanistic view-point, the most widely studied interaction types in interspecies cross-talks include (a) microbial metabolite-mediated networks, (b) protein–protein interactions (PPIs), and (c) RNA-mediated interactions. Accordingly, many of the computational methods developed to investigate microbe-host interactions have focused on the three above-mentioned interaction types (Figure 1). As a fourth method approach, integrated pipelines combine multiple microbial and host -omic data types and networks to infer the cumulative functional effects of inter-species interactions/communication on the host.

### Approaches Inferring Mechanistic Metabolic Interactions

The metabolomic layer (which comprises the enzymes, metabolites, and the reactional interactions between them) has a prominent influence on both health and disease states associated with alterations in microbiota composition (Wong et al., 2016; Martinez et al., 2017). Metabolic networks can thus represent and capture the underlying mechanisms driving various phenotypes (Pey et al., 2013; Samal et al., 2017; Zampieri et al., 2019). Computational approaches aimed at inferring the microbe-host co-metabolic networks can be classified into three prominent categories namely (a) Community-wide metabolic network modeling using metagenomic datasets: this approach is based on the assumption that the metagenomic read-outs represent the gene-distribution structure of the entire microbial community. The autonomy of species – i.e., information about which gene is derived from which species, are disregarded. Thus, the metabolic network reconstructed using this approach consists of relationships (reactions) catalyzed by enzymes (encoded by the measured genes) between molecular entities (metabolites) at a community level. (b) High throughput data driven approaches using metabolic datasets – this data-driven methodology uses targeted or untargeted profiling of metabolites from different groups of samples. Subsequently, multi-variate modeling methods and various statistical methods including simple PCAs are applied to identify biomarkers which distinguish different sample groups from each other. (c) Genome scale reconstruction applying constraint-based modeling approaches which are described below. The first two methods do not provide direct mechanistic insights and hence are not covered further in this review.

Genome-scale reconstruction models provide mechanistic information by integrating multiple inputs. These inputs include the curated genome scale metabolic models of both the host and microbial species, high-throughput meta -omic datasets including metabolites, reaction fluxes, biochemical traits and accessory phenotypic data. However, due to the strenuous nature of various steps involved in constructing the models and in scaling it up to multiple species or multiple hosts, only a handful of studies have applied this concept to infer microbe-host co-metabolic interactions (Table 1). The AGORA (assembly of gut organisms through reconstruction and analysis) collection is a resource of genome-scale metabolic models for 773 human gut bacterial species using a combination of metagenomics and experimental data from literature. Furthermore, the framework employed by AGORA is amenable to scale-up given its easy adaptability to novel species of interest. AGORA also serves as a source of genome scale metabolic models reconstructed in a standardized manner. Thus, various studies have in turn used the genome scale models from the AGORA resource to construct context-specific models (Bauer et al., 2017; Bunesova et al., 2018; Tramontano et al., 2018; Pryor et al., 2019; Yilmaz et al., 2019). Recently, the authors of AGORA and their collaborators extended the framework to 7206 strains by incorporating information on the drug-metabolizing potential of the bacterial strains (Heinken et al., 2020).

**TABLE 1:** Studies using genome-scale metabolic models and constraint based approaches to infer mechanistic co-metabolic interactions between microbial and host species.

Study	Context
Rodenburg et al. (2019)	Integrated metabolic model of *P. infestans* infecting tomato (*S. lycopersicum*)
Islam et al. (2019)	Genome-scale metabolic model between key members in the rumen microbiome and the viral phages
Hertel et al. (2019)	Integrated constraint-based model revealing microbe-host interactions in Parkinson’s Disease
Aller et al. (2018)	Genome-scale model integrating biochemical demands arising from virus production and human macrophage cell metabolism
Ding et al. (2016)	Simulation of co-metabolic model of different enteropathogens in response to various host environments
Heinken and Thiele (2015)	*In silico* microbe-host gut co-metabolic model to predict effects of different host dietary schemes
Heinken et al. (2013)	Experimentally validated gut co-metabolic model between commensal bacterium *B. thetaiotaomicron* and mouse
Bordbar et al. (2010)	*Francisella tularensis* infecting human alveolar macrophage supported by high-throughput data from infected conditions

The reported studies on genome-scale reconstruction models have been distributed across many different ecological contexts such as the human and rumen gut ecosystems (Islam et al., 2019), microbe-plant interactions, human alveolar macrophages, the effect of viral demands on the metabolism of human macrophages, microbe-host interactions in Parkinson’s Disease to name a few. Due to the mechanistic nature of such models, they can be used as a template for further integrating other -omic datasets. This not only refines the models thereby increasing their predictive power but also assigns contextuality.

By incorporating the individual reconstructed metabolic models of tomato (*Solanum lycopersicum*) and the tomato late blight pathogen *Phytophthora infestans*, Rodenburg et al. (2019) pointed out specific pathways which mediate the dependencies of the pathogen on the metabolism of *S. lycopersicum*. The individual metabolic models for *S. lycopersicum* and *P. infestans* were derived by manually adding reactions and sub-cellular localization of metabolites and reactions (based on curation of literature) to the corresponding genome-scale models. Furthermore, by over-laying dual RNA-seq transcriptomic datasets from the host-pathogen duo into the co-metabolic network, various metabolic changes characterizing the scavenging nature of *P. infestans* were revealed. A similar study was performed in a mammalian setting wherein co-metabolic interactions and metabolic exchanges were inferred between the respiratory pathogen *Mycobacterium tuberculosis* and human alveolar macrophages (Bordbar et al., 2010). The metabolic model for the alveolar macrophages was derived from Recon1, the global human metabolic model (Thiele et al., 2013b). Briefly, a curated version of Recon1 was overlaid with gene expression data for healthy, inactivated alveolar macrophages and combined with information on flux limits for major pathways of central metabolism and a host of heterogeneous datasets such as immunohistological staining, transporter proteins, etc (Bordbar et al., 2010). The macrophage model was then combined with that of *Francisella tularensis* and corrected for compartment-specific reactions and metabolites. Unsurprisingly, given the advancement in terms of data generated and metabolic models made available, most of the genome-scale metabolic reconstruction studies (Table 1) were carried out for the gut ecosystem (Heinken et al., 2013; Heinken and Thiele, 2015; Ding et al., 2016; Islam et al., 2019).

Other microbe-host co-metabolic studies have been performed using publicly available tools based on constraint-based modeling approaches. The Constraint-based reconstruction and analysis (COBRA) toolbox (Heirendt et al., 2019) is one such compendium of methods containing various user-guided steps to reconstruct genome-scale metabolic models. It is characterized by properties such as interoperability, customized reconstruction, modeling, visualization, modeling, simulation, and integration of -omic datasets in various contexts (compartments, cell-types, etc.). By harnessing these properties, researchers have used the COBRA toolbox to model and investigate microbe-host metabolic interactions (Heinken et al., 2013; Thiele et al., 2013a) in the context of mammalian health with implications on human health. A representative study of the gut ecosystem using the COBRA toolbox integrated two previously published constraint-based models of mouse and a gut commensal *Bacteroides thetaiotaomicron* (Heinken et al., 2013). The *B. thetaiotaomicron* model was generated by the manual curation of a seed model produced by Model Seed (Henry et al., 2010) from the genome sequence annotated using RAST (Aziz et al., 2008) (which is a prokaryotic genome annotation tool). The mouse metabolic model was compiled by integrating a previously annotated and reconstructed model with gene essentiality data from experiments followed by corrections for duplicate reactions. The two models were then brought together by setting rules based on the subcellular localization of metabolites and reactions. The integrated metabolic model could capture many of the phenotypes exhibited *in vivo* namely the dependence of *B. thetaiotaomicron* on glycans derived from the metabolism of the host as well as the host diet itself (Heinken et al., 2013). It is noteworthy to mention that the authors also introduced novel methodologies such as Pareto analysis to complement the power of the COBRA toolbox. Pareto analysis is a bi-objective linear programming-based methodology which enables the analysis and identification of growth dependencies and trade-offs between the microbe and the host as captured by their metabolic networks.

A similar study (Hertel et al., 2019) was performed using the COBRA toolbox in conjunction with other supplementary tools such as the Microbiome Modeling Toolbox (Baldini et al., 2019) which can integrate the individual reconstructed models together into one reconstructed model in addition to other useful properties (such as inferring interactions by taxa, reconstruction of pairwise/community co-metabolic networks, compartment-based modeling, pareto analysis, and various downstream operations) to extend the constraint-based modeling framework. The study integrated the microbiome and longitudinal metabolomic datasets from patients with Parkinson’s disease (Hertel et al., 2019). This microbiome-host -omic integration study provided clues as to how alterations in particular co-metabolized pathways (by both the host and microbiome) such as sulfur metabolism could contribute to the varying severity of the disease. In particular, the authors were able to identify that changes in the co-metabolized pathways could be driven by particular members of the gut microbiota. This opens up possibilities to design gut microbiome-based therapies to treat or even prevent Parkinson’s disease.

### Approaches Inferring Protein–Protein Interactions (PPIs)

Protein–protein interactions are one of the most well-studied interaction types mediating inter-species communication (Schweppe et al., 2015). Accordingly, a large number of computational microbe-host interaction studies have focused on PPIs. Congruently, PPI-based approaches have also been propelled by the adoption of concepts from other domains of computational biology and computational sciences in general. Hence, PPI-based approaches can be sub-classified into four predominant methods (Table 2) depending on the concepts used (1) Machine learning based PPI methods, (2) Structural feature based PPI methods, (3) Data/Literature mining based PPI methods, and (4) Interolog based PPI methods. In this section, we provide a brief overview of the concepts involved in each of these methods (Table 2) and provide a few representative examples.

**TABLE 2:** Computational approaches and methods inferring protein–protein interactions mediating inter-kingdom cross-talk between microbial and host organisms.

Method and corresponding studies	Reported use-case (host-microbe)
**Machine learning based methods**	
Leite et al. (2018)	Bacteria–phage
Tastan et al. (2009); Qi et al. (2010), Dyer et al. (2011); Nouretdinov et al. (2012), Shoombuatong et al. (2012); Mei (2013), Hongjaisee et al. (2019)	Human–HIV
Kshirsagar et al. (2013)	Human–*F. tularensis*, Human–*Y. pestis*, Human–*B. anthracis*, Human-*S. typhi*
Wuchty (2011)	Human–*Plasmodium falciparum*
Kösesoy et al. (2019)	Human–*Y. pestis*, Human–*B. anthracis*
Cui et al. (2012); Emamjomeh et al. (2014), Kim et al. (2017)	Human–Hepatitis C virus
HOPITOR (Basit et al., 2018)	Generic (Human–virus PPIs)
Liao et al. (2011)	Human–*Schistosoma japonicum*
Mei et al. (2018); Sun et al. (2018)	Human–*Francisella tularensis*
Kargarfard et al. (2016)	3 hosts and 674 influenza strains
Cui et al. (2012); Dong et al. (2015), Kim et al. (2017)	Human–Human papillomavirus
Lai et al. (2012)	Human–Influenza A virus
Mei and Zhu (2014a)	Human–HTLV retroviruses
Mei and Zhu (2014b)	Human–*Salmonella*
Lian et al. (2019)	Human–*Y. pestis*
**Structural feature based methods (features used)**	
Dyer at al. (2007) (DDI)	Human–*Plasmodium falciparum*
Nourani et al. (2016) (DDI)	Human–multiple viruses
Sudhakar et al. (2019) (DDI and DMI)	Human–multiple bacterial pathogens
Doolittle and Gomez (2011) (PSS)	Human–Dengue virus, *Aedes aegypti*–Dengue virus
Cui et al. (2016) (PSS)	Human–HIV, Human–*Francisella tularensis*
P-HIPSTer (Lasso et al., 2019) (PSS)	Human–multiple viruses
Chen at al. (2019) (PSS)	Human–Dengue virus 2, Human–West Nile virus
Guven-Maiorov et al. (2017) (Mimicry)	Human–*Helicobacter pylori*
Mahajan and Mande (2017) (DDI)	Human–*Francisella tularensis*
Zhang et al. (2017a) (DMI)	Grass carp–Grass carp reovirus
Mehrotra et al. (2017) (PSS, DDI, and localization)	Human–*Leptospira interrogans*, Human–*Leptospira biflexa*
Halehalli and Nagarajaram (2015) (DDI, DMI)	Human–multiple viruses
SugarBindDB (Mariethoz et al., 2016) (glycan mediated PPIs)	Generic
Rajasekharan et al. (2013) (PSS)	Human–Chandipura virus
Carducci et al. (2010) (DDI)	Human–papillomavirus type 16
Franzosa and Xia (2011) (PSS and sequence identity)	Human–multiple viruses
Sahu et al. (2014) (DDI)	Arabidopsis-*Pseudomonas syringae*
Zhou et al. (2018) (DDI)	Human–Dengue virus, *Aedes aegypti*–Dengue virus
Kim et al. (2017) (DDI)	Human–multiple viruses
Kerr et al. (2015) (Computational docking)	Human–Dengue virus 2, Human–West Nile virus
Evans et al. (2009) (DMI)	Human–HIV
Doxey and McConkey (2013) (Mimicry)	Human–*Francisella tularensis*
Mei and Zhang (2020) (Mimicry)	Human-*S. typhimurium* and Human-Human respiratory syncytial virus
**Data/Literature mining based methods**	
Thieu et al. (2012)	Generic
Viruses.STRING (Cook et al., 2018)	319 hosts and 239 viruses
Li et al. (2018)	Human–Epstein-Barr virus
Saik et al. (2016)	Human–Hepatitis C virus
García-Pérez et al. (2018)	Human–Influenza A virus
**“Interolog” based methods**	
Krishnadev and Srinivasan (2008); Lee et al. (2008)	Human–*Plasmodium falciparum*
Krishnadev and Srinivasan (2011)	Human–*E. coli*, Human– *S. typhimurium*, Human–*Y. pestis*
Tyagi et al. (2009)	Human–*Helicobacter pylori*
Cui et al. (2016)	Human–HIV, Human–*Francisella tularensis*
Schleker et al. (2012)	Human–*Salmonella, Salmonella*–*A. thaliana*
Li et al. (2012)	*A. thaliana*–*Ralstonia solanacearum*
Wallqvist et al. (2017)	Human–*Coxiella burnetii*
Cuesta-Astroz et al. (2019)	Human and 15 eukaryotic parasites
Zhou et al. (2014); Cui et al. (2016)	Human–*Francisella tularensis*
Barh et al. (2013)	Human–*Corynebacterium pseudotuberculosis*, Human–*Corynebacterium diphtheriae*, Human–*Francisella tularensis*, Human–*Corynebacterium ulcerans*, Human–*Y. pestis*, and Human–*E. coli*

*DDI, domain–domain interaction; DMI, domain-motif interaction; PSS, pairwise structural similarity. Supplementary Table 1 provides further details into the novelty of the methods and results.*

#### Structural Feature Based PPI Methods

Interactions between proteins are usually a by-product of physical interactions between structural features of the proteins and/or could be characterized indirectly by co-occurring functional features of the proteins (Ding and Kihara, 2018). Structural features of the proteins include their domain and motif architectures/compositions, amino acid composition and frequencies, post-translational modification signatures, amino acid k-mers, mimicry motifs and 3D structural properties (Ding and Kihara, 2018). Structural feature-based PPI prediction, applied initially for intra-species PPIs, was subsequently extended to inter-species studies. Essentially, the fundamental principle on which structural feature-based PPI prediction methods work involves the use of mechanistic evidence between structural features to identify potentially interacting proteins. These could include for example interactions between domains, between domains and motifs, post-translational modifications and pairwise structural similarity (Ding and Kihara, 2018). Such structural studies have been confined to considerably well studied species pairs involving H. sapiens and prominent viral and bacterial pathogens (Table 2). Along with pairwise structural similarity-based methods using 3D protein complexes, domain–domain interaction (DDI) and domain-motif interaction (DMI) based methods are one of the most commonly used methods within the structural feature based methodological framework for predicting inter-species PPIs. Due to the ease of annotating domains and motifs, DDI- and DMI-based methods have been harnessed widely (Table 2). While DDI based methods have been applied to infer PPIs for a large number of species-pairs including Human–*Plasmodium falciparum* (Dyer et al., 2007), Human–*Francisella tularensis* (Zhou et al., 2013; Mahajan and Mande, 2017), Human–*Leptospira interrogans* (Mehrotra et al., 2017), Human–*Leptospira biflexa* (Mehrotra et al., 2017), Human–papillomavirus type 16 (Carducci et al., 2010), Arabidopsis–*Pseudomonas syringae* (Sahu et al., 2014), Rice–*Xanthomonas oryzae* (Kim et al., 2008), they have the inherent disadvantage of not being able to explicitly discern directionality.

**FIGURE 2:**
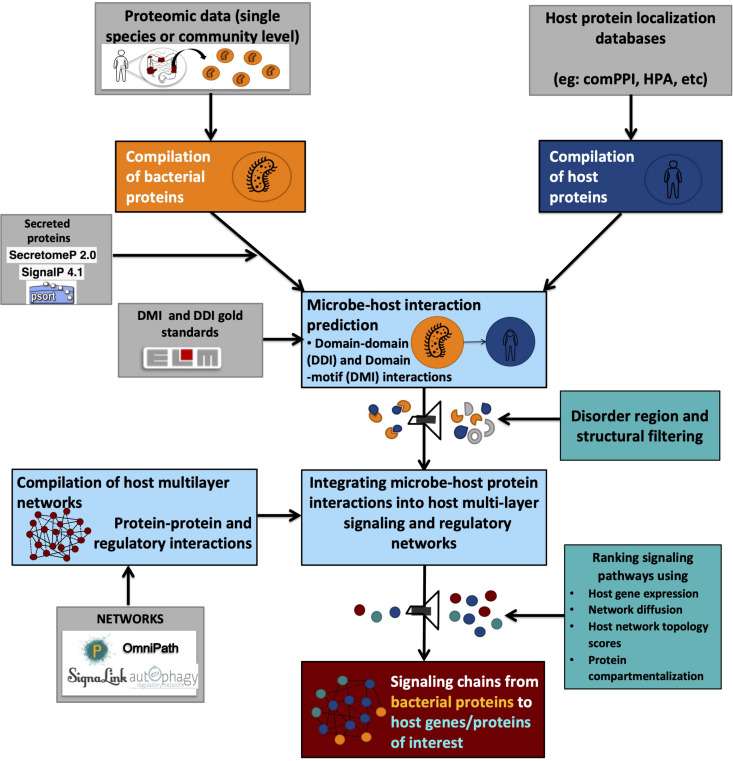
Graphical representation of a typical integrated workflow predicting interactions between microbial and host proteins and their effect on host processes.

On the other hand, DMIs provide directionality for PPIs, thus indicating the flow of signal transduction (Akiva et al., 2012; Gibson et al., 2015). For example, if a microbial protein A contains a domain known to be interacting with a motif on the host protein B, it is graphically represented as A > B, translating into “microbial protein A modulates host protein B.” Due to their specificity, DMI-based methods are preferred over DDI based methods for research questions seeking to answer the role of post-translational modifications elicited on host proteins by microbial proteins or vice versa. However, due to the short sequence length of protein sequence motifs, even the most stringent search strategies have the tendency to result in thousands of false-positive hits while performing motif searches on a proteome-wide basis (Perkins et al., 2010; Idrees et al., 2018). Therefore, proper quality controls need to be applied to filter out false-positives based on structural properties such as the occurrence of truly interacting motifs within disordered regions and outside globular domains (Perkins et al., 2010; Idrees et al., 2018; Figure 2).

Several studies (Table 2) have been conducted to apply the principles of DMIs to predict PPIs for multiple microbe-host species-combinations including grass carp-grass carp reovirus (Zhang et al., 2017a), human-multiple bacterial pathogens (Sudhakar et al., 2019) and human-multiple viruses (Evans et al., 2009; Halehalli and Nagarajaram, 2015). By integrating DMI predictions between grass carp and grass carp reovirus (GCRV) proteins with differential gene expression and tissue-specific gene expression followed by functional enrichment, Zhang et al. (2017a) were able to pinpoint several signaling pathways modulated by GCRV. The authors also highlight an enrichment of host genes expressed in the intestinal niche suggesting that GCRV might have a higher influence on the gut. Recently, we conducted a study (Sudhakar et al., 2019) using DDI and DMI based methods to identify cross-talks between several bacterial pathogens including *Salmonella* and autophagy – a prominent biological process involved in host cellular homeostasis. Firstly, to identify microbial proteins targeted by selective autophagy, we scanned the bacterial proteins for the presence of the recognition motifs corresponding to the selective autophagy receptors p62 and NDP52 and the autophagy adapter protein LC3. Conversely, to infer the modulation of host autophagy by the bacterial pathogens, DMI and DDI based methods were used to identify the bacterial proteins which are able to bind to/modulate the 37 core autophagy host proteins. By overlapping the two above-mentioned sets of predictions, bacterial proteins involved in interplays were identified. Such bacterial proteins are also targeted by the host autophagy machinery for clearance and degradation. This was followed by experimentally verifying the effect on autophagy of a *Salmonella* protease involved in human-*Salmonella* interplay.

A variation of the motif-based methodologies is the use of motifs to characterize pathogen mimicry. This essentially involves the identification of eukaryotic linear motifs on microbial proteins which in turn can hijack host proteins and thereby promote antagonistic binding (Hurford and Day, 2013; Via et al., 2015). Motif-mediated molecular mimicry therefore rewires the host signaling and regulatory networks by titrating essential host proteins and enabling the microbe to create favorable micro-environments in the host cell by altering immune responses for example (Cusick et al., 2012). In addition to motifs, molecular mimicry can also be mediated at the level of protein, structural and interface levels. At the protein level, specific studies investigating the role of molecular mimicry in the pathogenesis of prominent bacterial pathogens (Doxey and McConkey, 2013) including *Salmonella typhimurium* and *Human respiratory syncytial virus* (Mei and Zhang, 2020) have been carried out (Table 2). At the interface level, Guven-Maiorov et al. (2017) devised a computational method to infer mimicry induced by a prominent gastric cancer causing pathogen *Helicobacter pylori*. Besides DDI and DMI based methods, researchers have also used other structure-based methodologies such as pairwise structural similarity (PSS) to predict inter-species PPIs. PSS methods at their very core are based on the premise that proteins possessing similar structures have a greater probability of interacting with the same set of protein partners (Ding and Kihara, 2018). This has been applied to infer the interactions with the host of various pathogens such as Dengue virus (Doolittle and Gomez, 2011), HIV (Cui et al., 2016), *Francisella tularensis* (Cui et al., 2016), West Nile virus (Chen et al., 2019), Chandipura virus (Rajasekharan et al., 2013), and other viral pathogens (Franzosa and Xia, 2011; Lasso et al., 2019).

As a means of ensuring proper quantitative evaluation of *de novo* PPI predictions, emerging computational methods such as machine learning have been used in conjunction with structural-feature based PPI prediction methods. In order to avoid repetitions, methods using ML for evaluating the performance of structural feature dependent PPI predictions are discussed in the next subsection.

#### Machine Learning Based PPI Methods

Due to their ability to discern complex patterns among a large number of features in big datasets, machine learning (ML) methods have found favor in various applications of computational biology and bioinformatics (Shastry and Sanjay, 2020) including the prediction of microbe-host molecular interactions. A variety of supervised and unsupervised methods have been used to predict the interactions between microbial and host proteins (Table 2). In general, supervised machine learning methods utilize features from “gold-standard” interaction datasets to identify potential protein–protein interaction pairs from the user provided list of microbial and host proteins (Zhang et al., 2017b). In supervised methods, the “gold-standard” datasets are either compiled from high-throughput experimental methodologies or from curated lists of interactions from the literature (Zhang et al., 2017b). In the case of ML being used in combination with “interolog” based methods (explained in section 5.2.4), “gold-standard” PPI datasets can also be retrieved from other related or unrelated microbe-host species pairs depending on the scope of the study. Some of the features used to infer *de novo* PPI predictions include protein properties such as post-translational modifications, chemical composition, tissue distribution, molecular weight, domain/motif compositions, ontologies, gene expression, amino-acid frequencies, homology to human binding partners, and relevance of proteins in host network. By using these features, supervised methods are able to discern truly interacting protein pairs from all possible pairs of microbial and host proteins (Zhang et al., 2017b).

Supervised methods can also be differentiated by the kind of ML methodology/model used for the task of rightly classifying truly interacting protein pairs. Several supervised studies employing individual ML models [such as I2-regularized logistic regression (Mei et al., 2018), random forests (RF) (Kösesoy et al., 2019), etc], support vector machine (SVM) (Cui et al., 2012; Shoombuatong et al., 2012; Kim et al., 2017) have been applied to infer PPIs between microbial and host species. SVMs use a framework of searching and finding the best hyperplane (aka decision boundary represented by a mathematical equation) to separate sample with different labels corresponding to a class. Several variations of the SVM exist to handle data with underlying linear or non-linear relationships (Byvatov and Schneider, 2003).

Using four different ML models namely RF, SVM, Artificial Neural Networks (ANN) and K-Nearest Neighbors (K-NN), and multiple lines of -omic evidence including experimental PPIs as predictive features, Leite et al. (2018) devised a model based on a supervised protocol to accurately predict bacterium-phage interactions. The model, a type of ensemble learning, due to its generic nature, can also be used to predict interactions between any two given species, given the availability of informative feature sets. Ensemble learning (Che et al., 2011), combines multiple individual classifiers to achieve a final classification and has been used to predict PPI based HIV-human and hepatitis C virus-human networks (Mei, 2013; Emamjomeh et al., 2014). Ensemble classification methods outperform individual classifiers based on several use-cases (Krawczyk, 2015; Haque et al., 2016; Yijing et al., 2016; Lin et al., 2019) and can be generalized into three distinct categories namely bagging, boosting and stacked generalization. The last of the three approaches, stacked generalization, was used by Emamjomeh et al. (2014) to predict PPIs between human and the hepatitis C virus. While bagging assigns training sets to individual classifiers based on a random selection of the initial training dataset with replacement for subsequent sampling runs, boosting involves the creation and evaluation of classifiers in a sequential manner, with the succeeding classifier assigning more weights to the misclassification errors committed by the preceding classifier. The “boosted” weights are then normalized for all the instances in the entire dataset which is then used as the training dataset for the next classifier after which the final classification step is carried out based on the weighted individual classifiers. The stacked generalization methodology is designed to overcome some of the errors committed by the individual classifiers even if they are used in the ensemble framework. The stacked approach achieves this by using a “stacks” of base learners so that its output is the input for a meta-learner which knows how best to combine the base learners’ outputs. The training data may or may not overlap between the two stacks and can be specified accordingly.

Various auxiliary algorithms have been used in conjunction with machine learning methods to predict inter-species PPIs. An example of such a study includes the use of a novel protein sequence based feature extraction method called Location Based Encoding (LBE) with different classifier models including RFs. Such integrated methodologies have been used to predict protein interactions with the human host of two important pathogens – *Bacillus anthracis* and *Yersinia pestis* (Kösesoy et al., 2019). LBE is a methodology which complements the ML approaches for PPIs by differentiating proteins only based on the locations of the amino acids in the sequence (Li et al., 2009).

Supervised methods are sometimes constrained due to the small size of “gold-standard” datasets that restricts the inference and prediction of proteome-wide PPIs between the full list of proteins of any two given species. Mei and Zhu (2014a) harness the power of multi-instance AdaBoost, a type of boosting-based ensemble learning protocol, which is a multi-instance learning based ML method, to reconstruct proteome-wide Human T-cell leukemia virus-human PPI networks using homology knowledge derived protein features. AdaBoost improves classification performance by combining multiple weak classifiers into one strong classifier. It works in part by assigning more weight to instances which can only be classified with greater difficulty than to instances which can be easily classified (Kim et al., 2012). The dearth of true interacting protein-pairs has also prompted researchers to use unsupervised or semi-supervised approaches to infer microbe-host PPIs. Qi et al. (2010) complement the list of true interactions with a list of protein-pairs wherein association evidence exists with no interaction evidence between the proteins of a pair. Supervised learning is performed thereafter with a multilayer perceptron network and by using the true interaction list. Subsequently, the semi-supervised approach uses the same network layers of the supervised classifier but instead trains on the protein-pairs with association evidence only. By using this hybrid approach, the authors report improved performance for predicting interactions between HIV and human proteins (Qi et al., 2010).

#### Data/Literature Mining Based PPI Methods

Even though many databases have been compiled to collect, curate and store microbe-host PPIs (Kumar and Nanduri, 2010; Durmus Tekir et al., 2013; Cook et al., 2018; Gao et al., 2018; Singh et al., 2019), these are mostly confined to well-studied pathogens and are predominantly comprised of interactions from high-throughput experiments. Contrastingly, in the literature, there exist inter-species PPIs from low-throughput experiments with some of them from non-model organisms, and commensal microbes, but mostly distributed over several individual studies. Very often, the inter-species PPI databases and repositories do not capture these sparse interactions. Hence, researchers have adapted and modified data- and text-mining tools to search for and extract microbe-host PPIs from existing literature. Retrieving such PPIs not only helps in increasing the number of true positive and true negative interactions (which helps aid the predictive performance of algorithms) but also extends our knowledge of existing microbe-host interactions. Motivated by the above explained need to mine-out microbe-host PPIs, Thieu et al. (2012) combine and compare the performance of a language based method based on a link grammar parser to a supervised ML methodology (SVM) and report that the combined approach results in a higher classification accuracy when compared to existing literature mining methods. As part of a bigger analytical framework aimed at uncovering the cellular mechanisms involved in human B lymphocytes during *Epstein-Barr virus* infection, Li et al. (2018) use a big-data mining methodology to identify a diverse range of inter-species molecular interactions including PPIs. Similar text/data mining approaches were also executed to extract PPI-mediated interactions of the human host with multiple viruses such as Hepatitis C virus (Saik et al., 2016) and Influenza A virus (García-Pérez et al., 2018; Table 2).

#### Interolog Based PPI Methods

For most species-pairs of interest, especially those belonging to the category of non-model organisms, there is a scarcity of experimentally verified PPIs. This has necessitated the development of novel bioinformatic methods, one of which is the inference of interactions from existing experimentally determined inter-species PPIs (Kshirsagar et al., 2015). These types of methodologies are usually based on the principle of homology (hence the term “interolog”: meaning interacting orthologs) – either at the level of proteins or protein structural features or both. Protein features used for homology based extrapolation include but are not limited to domains, motifs, amino-acid k-mers, and 3D structural properties (Kshirsagar et al., 2015). Interolog based approaches have been applied to harness the large volume of experimentally verified PPIs for model organisms including prominent bacterial/viral pathogens. Despite the potentially large coverage that can be achieved by such approaches, there exist several disadvantages of using interolog approaches as a silver bullet for inferring inter-species PPIs especially for novel species-pairs. These disadvantages are attributed to different pathogenic mechanisms between the microbes in the context of infecting different host species, different cellular localizations, and varying activity levels (expression, post-translational modifications, etc.) of the orthologous microbial proteins. Such differences lead to accessibility bottlenecks i.e., the ability of the proteins to physically access host proteins and thereby interact. Hence, interolog based approaches need to be complemented with additional filtering and quality control steps such as selecting proteins from infection-relevant cellular compartments, expression/activity measurements, etc.

Interolog based methods have been used to infer inter-species PPIs for many prominent pathogens and parasites (Table 2). Different versions of the interolog approach have been used to extrapolate PPIs corresponding to interactions between the human host and various pathogens such as *Plasmodium falciparum* (Krishnadev and Srinivasan, 2008; Lee et al., 2008), *Escherichia coli* (Krishnadev and Srinivasan, 2011), *S. typhimurium* (Krishnadev and Srinivasan, 2011; Schleker et al., 2012), *Y. pestis* (Krishnadev and Srinivasan, 2011), *Helicobacter pylori* (Tyagi et al., 2009), HIV (Cui et al., 2016), *Francisella tularensis* (Zhou et al., 2014; Cui et al., 2016), *Coxiella burnetii* (Wallqvist et al., 2017), *Corynebacterium pseudotuberculosis* (Barh et al., 2013), *Corynebacterium diphtheriae* (Barh et al., 2013), and *Corynebacterium ulcerans* (Barh et al., 2013). Using PPIs from the STRING database as the starting interaction set, Cuesta-Astroz et al. (2019) used the interolog methodology to predict PPIs between 15 different eukaryotic pathogens and the human host. To assign species-specific and lifecycle- specific contextuality, the authors confined the analysis to proteins from particular cellular compartments which are relevant to the infection process. From the analysis of the ensuing PPI networks, various invasion and evasion mechanisms adopted commonly and specifically by particular parasites were inferred (Cuesta-Astroz et al., 2019). Schleker et al. (2012) present another version of the interolog approach to predict human-*Salmonella* and *A. thaliana-Salmonella* PPI networks. As a source of template PPIs, publicly available interaction databases are used along with databases containing 3D structures between Pfam domains. As an add-on to the sequence based orthology of proteins, domain based orthology is also performed in order to reduce the false positive rates. Several additional filtering strategies such as restriction to predicted transmembrane proteins, relevance in host network and functional attributes such as gene ontology are used to make the PPIs more specific.

### Approaches Inferring RNA Mediated Interactions

The role of RNAs, especially non-coding RNAs such as long non-coding RNAs (lncRNAs) and microRNAs (miRNAs) in mediating molecular microbe-host interactions have been reported in the literature (Li et al., 2015b; Agliano et al., 2019). RNA molecules are either secreted by the microbial cell into the host cell or are packaged into vesicles along with other molecules which are then taken up by the host cell by endocytosis (Weiberg et al., 2014; Huang et al., 2019; Ahmadi Badi et al., 2020). Such microbial RNAs then modulate host cell activity by either binding to DNA, messenger RNAs or proteins. Thus, by salvaging and titrating host components, microbial RNAs modulate regulatory and signaling networks and subsequently host cell activity (Duval et al., 2017; Agliano et al., 2019; Shirahama et al., 2020). However, in contrast to PPI based methods, even though RNA-mediated microbe-host interactions are well studied from an experimental point of view, very few methods or studies exist that have systemically and systematically applied computational analysis (Table 3). As such, the resources which exist in the domain of RNA-mediated microbe-host interactions comprise of databases such as ViRBase (Li et al., 2015b) which is predominantly a source of experimentally verified virus–host non-coding RNA-associated interactions. In addition, it also contains predicted binding sites of virus non-coding RNAs on host proteins and RNAs. A prominent study which comprehensively examines and evaluates the role of RNAs in microbe-host interactions is that of Saçar Demirci and Adan (2020) who investigated the roles in infection of miRNA-like sequences encoded within the Severe Acute Respiratory Syndrome Coronavirus 2 (SARS-CoV-2) genome. They used a modified version of izMiR (Allmer et al., 2016), a SVM based ML method to predict pre-miRNAs which are homologous to the human precursor miRNAs from miRbase. The SVM based ML method identified several viral hairpin sequences which were smaller in length compared to the human miRNA precursors while many of the human and viral miRNA precursors were similar in length and shared identical minimum free energy, a feature used by the izMiR workflow (Allmer et al., 2016). Based on this observation, a revised classifier trained using only the known human miRNAs was used on the entire SARS-CoV-2 hairpin dataset which resulted in the identification of potential hairpins from which mature miRNA candidates were extracted. As a next step, the psRNATarget tool (Dai et al., 2018) was used to predict *de novo* the human genes targeted by the inferred viral miRNAs. Functional analysis of the human genes targeted revealed that the SARS-CoV-2miRNAs can affect various host processes including transcription, defense systems, Wnt and EGFR signaling pathways.

**TABLE 3:** Examples of studies utilizing computational approaches to infer RNA-mediated interactions between microbes and hosts.

Study	Context
Saçar Demirci and Adan (2020)	Analysis revealing the potential interactions between mature micro-RNA like viral RNA sequences and host genes
ViRBase (Li et al., 2015b)	Source of experimentally verified virus–host non-coding RNA-associated interactions; also contains predicted binding sites of virus non-coding RNAs on host proteins and RNAs

### Approaches Utilizing Pipelines Integrating Multiple-Omic Datasets

Besides the computational methods based on particular types of molecular interactions, some integrated pipelines (Table 4) have been compiled to infer mechanistic microbe-host interactions. In general, such pipelines (Figure 2) incorporate the prediction of at least one molecular interaction type between microbial and host molecular components followed by various other functionalities such as integration of host responses. Table 5 provides a non-exhaustive overview of the different tools, databases and resources which are available in the public domain to compile integrated workflows based on PPIs for example.

**TABLE 4:** Integrated pipelines used to infer microbe-host interactions by combining heterogeneous -omic datasets.

Methodology	Functionalities
MicrobioLink (Andrighetti et al., 2020)	Integrating microbe-host protein interaction networks with host responses and host regulatory/signaling networks using network diffusion principles
KBase (Arkin et al., 2018)	Integrated platform enabling data sharing, integration, and analysis of -omic datasets from microbes, plants, and their communities by creating computational workflows
Li et al. (2015a)	Identifying critical effectors involved in host-pathogen interactions by integrating multiple lines of -omic evidence

**TABLE 5:** A non-exhaustive catalog of resources, tools and databases to compile protein–protein interaction based workflows for inferring microbe (microbiome)-host interactions.

Step in workflow	Resource/Tool/Database
Source of proteomes (sequence information)	UniProt (The UniProt Consortium, 2018), HumanPSD (Hodges et al., 2002), YPD (Payne and Garrels, 1997), PombePD (Costanzo et al., 2001), WormPD (Costanzo et al., 2001), and SWISS-PROT (Bairoch and Apweiler, 1996)
Source of proteomic datasets (expression information)	ProteomicsDB (Schmidt et al., 2018), Human Protein Atlas (HPA) (Thul and Lindskog, 2018), PRIDE (Perez-Riverol et al., 2019), PeptideAtlas (Desiere et al., 2006), MassIVE.quant (Choi et al., 2020), jPOSTrepo (Okuda et al., 2017), iProX (Ma et al., 2019), and Panorama Public (Sharma et al., 2018)
Proteomic annotations (structural features)	InterPro (Mitchell et al., 2019), Pfam (El-Gebali et al., 2019), ELM (Gouw et al., 2018), and PDB (Burley et al., 2017)
Protein sub-cellular localization (databases and prediction tools)	ComPPI (Veres et al., 2015), HPA (Thul and Lindskog, 2018), LocDB (Rastogi and Rost, 2011), LocSigDB (Negi et al., 2015), COMPARTMENTS (Binder et al., 2014), eSLDB (Pierleoni et al., 2007), SCLpred-EMS (Kaleel et al., 2020), DeepLoc (Almagro Armenteros et al., 2017), PSORTdb (Peabody et al., 2016), SecretomeP (Bendtsen et al., 2004), and Signal P (Armenteros et al., 2019)
Base information for prediction of PPIs	Domain-domain predictions – DOMINE (Raghavachari et al., 2008) and Domain-motif predictions – ELM (Gouw et al., 2018)
Quality control of inferred PPIs (using disordered region prediction)	IUPred (Mészáros et al., 2018), PrDOS (Ishida and Kinoshita, 2007), D2P2 (Oates et al., 2013), PONDR-FIT (Xue et al., 2010), DISOPRED (Ward et al., 2004), MFDp2 (Mizianty et al., 2013), and Meta-Disorder (Kozlowski and Bujnicki, 2012)
Network resources	OmniPath (Türei et al., 2016), IntAct (Orchard et al., 2014), Reactome (Fabregat et al., 2018), STRING (Szklarczyk et al., 2017), HTRI (Bovolenta et al., 2012), and DoRothEA (Garcia-Alonso et al., 2018)
Network diffusion approaches	NBS (Hofree et al., 2013), HotNet (Vandin et al., 2011), TieDie (Basha et al., 2013; Paull et al., 2013), RegMod (Qiu et al., 2010), and stSVM21 (Cun and Fröhlich, 2013)
Databases for host gene expression	GEO (Clough and Barrett, 2016) and ArrayExpress (Parkinson et al., 2007)

KBase (Arkin et al., 2018) is an integrated bioinformatics platform enabling users to share datasets with the research community as well as facilitating the integration, and analysis of -omic datasets from microbes and plants by creating computational workflows. Recently, we developed MicrobioLink (Andrighetti et al., 2020), an integrated pipeline which carries out *de novo* DDI and DMI based microbe-host PPI prediction followed by quality control using information from disordered region predictions from built-in tools such as IUPred (Mészáros et al., 2018). The pipeline then utilizes network diffusion principles and tools (Paull et al., 2013) to infer the molecular mechanisms and signaling pathways which mediate the effect of microbial proteins on host responses as measured by transcriptomic or proteomic read-outs. Flexibility is provided for users to feed in the desired datasets at any given step of the pipeline. Given the advent of new computational tools in inter-species interactions and pipeline management platforms, it is expected that an increasing number of dedicated bioinformatic workflows for microbe-host interactions will be developed in the near future.

## Discussion: Opportunities and Challenges

### Opportunities

#### Clinical and Translational Research

Since the aforementioned computational tools help researchers narrow down on both microbial and host components involved in mechanistic cross-talks, the tools may discover molecules which can delineate different clinical phenotypes. In addition, they can also be possible targets for therapeutic interventions. In other words, mechanistic predictions combined with clinical meta-data have a dual-purpose – they provide information on molecular components which could both represent and drive clinical phenotypes (Younesi, 2015) and thereby could potentially minimize our reliance on association-based biomarkers alone which need not explain causality (Levenson and Mori, 2014). The discovery of such mechanistic knowledge warrants the combinatorial use of different methodologies including machine learning and molecular interaction analysis. While many community level studies have been conducted on meta -omic datasets for the clinical classification of patients and the discovery of associative biomarkers (Wen et al., 2017; Yu et al., 2020; Clos-Garcia et al., 2019; Conteville et al., 2019), they have not incorporated mechanistic inferences. On the other hand, most mechanistic studies (Tables 2, 3) have been carried out on particular pathogens/microbial species without including clinical meta-data and/or clinical classifications.

Multi-omic approaches integrating heterogeneous -omic datasets from patients have been implemented for several diseases including IBD (Lloyd-Price et al., 2019) which are associated with microbial dysbiosis. However, these studies do not provide the required mechanistic insights for formulating therapeutic interventions. Beltran and Brito (2019) devised an integrated methodology to unravel the molecular mechanisms underlying the microbe-host interactions associated with various diseases such as colorectal cancer, IBD, obesity and type-2 diabetes. The aforementioned study represents one of the first and few initiatives to use community-wide microbe-host interaction predictions using meta -omic datasets from patients to discover mechanistic interactions driving the clinical phenotypes. By combining orthology based approaches to extrapolate interactions from experimental PPIs, machine learning and patient derived -omic datasets, the authors identified a subset of inter-species PPIs which are associated with disease phenotypes (Beltran and Brito, 2019). Thiele et al. (2020) published a novel study by integrating different levels of information (dietary information, physiological parameters, organ weights, and organ connectivities, etc.) and datasets such as molecular -omics (proteomics, metabolomics, metabolites produced by the gut microbiota) in an organ specific manner to arrive at a whole-body-model of human metabolism. Although not fully mechanistic, with this model, the authors were able to predict biomarkers of inherited metabolic diseases and host-microbiome co-metabolism. Such integrated studies and workflows combining statistical and mechanistic inference of multi -omic datasets awaits further adoption and application in the research on various diseases associated with microbial dysbiosis.

#### Research on Comparative Ecological Networks

The tools and resources listed in this review can be used to infer and predict molecular interactions between species in several contexts [microbe/microbiota in host, microbe/microbiota in several hosts, microbe (vs) microbe, and microbiota (vs) microbe, etc]. In almost all of the above-mentioned cases, molecular interactions between the autonomous entities (be it species or communities) could be driving the emergent phenotypes. Since the tools discussed in this manuscript also concern themselves with extrapolating interactions based on homology between species-pairs, it could be a right fit to predict *de novo* interaction relationships for species with very little experimental interaction information.

For example, Crohn’s disease, a sub-type of IBD, is characterized by the dysbiosis of the gut microbiome (Joossens et al., 2011; Schaubeck et al., 2016; Shaw et al., 2016). This results in persistent inflammation of the gut mucosal barrier as a result of the unbalanced host responses (co-influenced by host genetic factors as well) to the dysbiosed microbiome and its various components such as proteins, metabolites, etc (Li et al., 2014; Lavelle and Sokol, 2020). Some of the CD patients also display lesions of the skin during or after therapeutic regimens (Huang et al., 2012; Gravina et al., 2016). It is known that the skin also houses a complex microbial community which plays a role in maintaining homeostasis (Schommer and Gallo, 2013; Chen et al., 2018). Understanding the mechanisms by which CD medications impact the microbe-host interactions in the gut as well as the skin could help in avoiding the unintended side-effects of therapy in CD.

Yet another relevant context to apply the tools discussed herein is the inference of underlying molecular mechanisms which mediate the evasion of immune responses by bacterial pathogens in various hosts and their importance in transmission between hosts. We recently showed that bacterial pathogens and autophagy, a primary intracellular line of defense in the host, are engaged in an evolutionary tug of war, as evidenced by the presence of various interplays and cross-talks (Sudhakar et al., 2019). Given the exposure of host animals such as poultry and cattle to xenobiotic compounds such as antibiotics, many zoonotic pathogens are under constant selection pressure to evolve survival strategies to modulate/evade/survive within the host animal (Harada and Asai, 2010). This opens the door for impending risks of transmission (from animal hosts to human hosts or between various animal hosts) via the food chain of zoonotic species which have been selected for survival over many generations of persistence in the host (Farrell and Davies, 2019; Mollentze and Streicker, 2020). Microbe-host interaction mechanisms are at the evolutionary cross-roads of such transmission events between hosts. In this context, studying such interactions is expected to provide deeper insights into designing strategies to prevent and/or minimize spill-over transmission events.

### Challenges

Over the past decade, various advances in the domain of computational analysis of microbe-host interactions have been made. However, despite this progress, there remain many challenges as described below. These challenges also present opportunities and the need to come up with innovative approaches and solutions.

#### Catching Up With Complex Infection Processes

Infection biology has taken new strides over the past years with new molecule classes (Katiyar-Agarwal and Jin, 2010; Rana et al., 2015; Duval et al., 2017; Long et al., 2017; Peters et al., 2019; Acuña et al., 2020) and cell-types (Chattopadhyay et al., 2018) being discovered as having a role in the infection process. With that, novel interaction types between various molecular classes are also unearthed (Silmon de Monerri and Kim, 2014). In some cases, computational methods have not caught up with molecular mechanisms. For example, hepadnaviruses utilize host DNA ligases to generate covalently closed circular DNAs which play a major role in mediating viral infection and persistence (Long et al., 2017). Similarly long non-coding RNAs are known to be involved in host-pathogen interactions (Duval et al., 2017; Agliano et al., 2019). However, till date, computational methods do not exist to predict or infer the mechanisms by which the viruses recruit the host DNA ligases or directly modulate the biogenesis, conformation and activity of long non-coding RNAs. Hence, computational method developments are always a step behind the complexity associated with infection biology. This gap is all the more prevalent for commensal organisms in contrast to pathogens due to the constant and historically prevalent study bias.

#### Lack of Experimental Datasets

Non-model organisms and non-pathogenic organisms such as probiotics and commensals also suffer from a considerable knowledge gap in terms of known/experimentally verified molecular interactions. This affects the performance of computational methods considerably due to the need for large sets of true positives for the satisfactory performance and assessment of predictive algorithms (Jiao and Du, 2016). In addition, this also influences the coverage and accuracy of interolog approaches since they harness already existing true positive datasets for extrapolating to the species-pairs of interest based on orthology.

#### False-Positives

As with any computational algorithm, microbe-host interaction prediction methods also face the curse of false positives. This issue could be exacerbated by the availability of relatively small true positive (truly interacting) and true negative (non-interacting sets) datasets (Jiao and Du, 2016). Furthermore, the evolutionary distance and difference in infection process between the template species-pairs and the species-pair of interest as well as the absence of orthologous molecular components involved in the interactions could also contribute to the inflated false positive rates, reduced performance and coverage.

#### Community-Wide Interaction Prediction

Most of the microbe-host interaction computational tools have been directed at uncovering interactions corresponding to individual microbe-host pairs. This is a major drawback of existing methodologies, especially given the fact that phenotypes related to health and disease are associated with changes in community wide alterations (Clemente et al., 2012; Koboziev et al., 2014; Wang et al., 2017; Bailey and Holscher, 2018; Dominguez-Bello et al., 2019).

#### Modeling Dynamics of Microbe-Host Interactions

Last but not the least, current methods involved in microbe-host interaction analysis are not equipped to handle the dynamic nature of natural ecosystems and ecological niches in which the interactions are embedded. Although it is a generic drawback of many bioinformatic approaches, this challenge will need coordinated efforts between modelers, experimental biologists and bioinformaticians.

## Conclusion

Since the advent and expansion of high-throughput sequencing technologies, various observational studies of microbial communities inhabiting various ecological niches (inside host organisms for example) have been carried out. This has mostly resulted in associations with health- or disease-associated phenotypes. However, there is a huge gap in terms of the mechanisms mediated by these microbial communities and how these mechanisms contribute to the observed phenotypes. Despite the availability of experimental datasets which capture some of these mechanisms such as PPIs, these are either confined to model organisms or well-studied pathogens. Computational approaches provide researchers with the tools to upscale microbe-host interaction research by enabling them to make *de novo* inter-species molecular interactions and to extrapolate existing microbe-host interaction datasets to the species-pairs of interest. Computational methods may aid the study of microbe-host interaction by reducing the variable space, prioritizing interactions, and eventually building hypothesis for further experimental verification.

## Author Contributions

PS performed the literature review and wrote the manuscript. KM provided critical feedbacks and contributed to the text. BV contributed to relevant discussion about the clinical implications. TK and SV supervised the work and provided valuable discussions, feedbacks, and comments. All authors contributed to the article and approved the submitted version.

## Conflict of Interest

BV received lecture fees from AbbVie, Ferring Pharmaceuticals, Janssen, R-Biopharm, and Takeda; consultancy fees from Janssen and Sandoz. SV: research grant: MSD, AbbVie, Takeda, Pfizer, and J&J; lecture fee: MSD, AbbVie, Takeda, Ferring, Centocor, Hospira, Pfizer, J&J, and Genentech/Roche; consultancy: MSD, AbbVie, Takeda, Ferring, Centocor, Hospira, Pfizer, J&J, Genentech/Roche, Celgene, Mundipharma, Celltrion, SecondGenome, Prometheus, Shire, ProDigest, Gilead, and Galapagos. SV is a senior clinical investigator of the Research Foundation–Flanders (FWO). The work of TK was supported by BenevolentAI and Unilever. The remaining authors declare that the research was conducted in the absence of any commercial or financial relationships that could be construed as a potential conflict of interest.

## Supplementary Material

Supplementary Table 1 Studies using genome-scale metabolic models and constraint based approaches to infer mechanistic co-metabolic interactions between microbial and host species.
